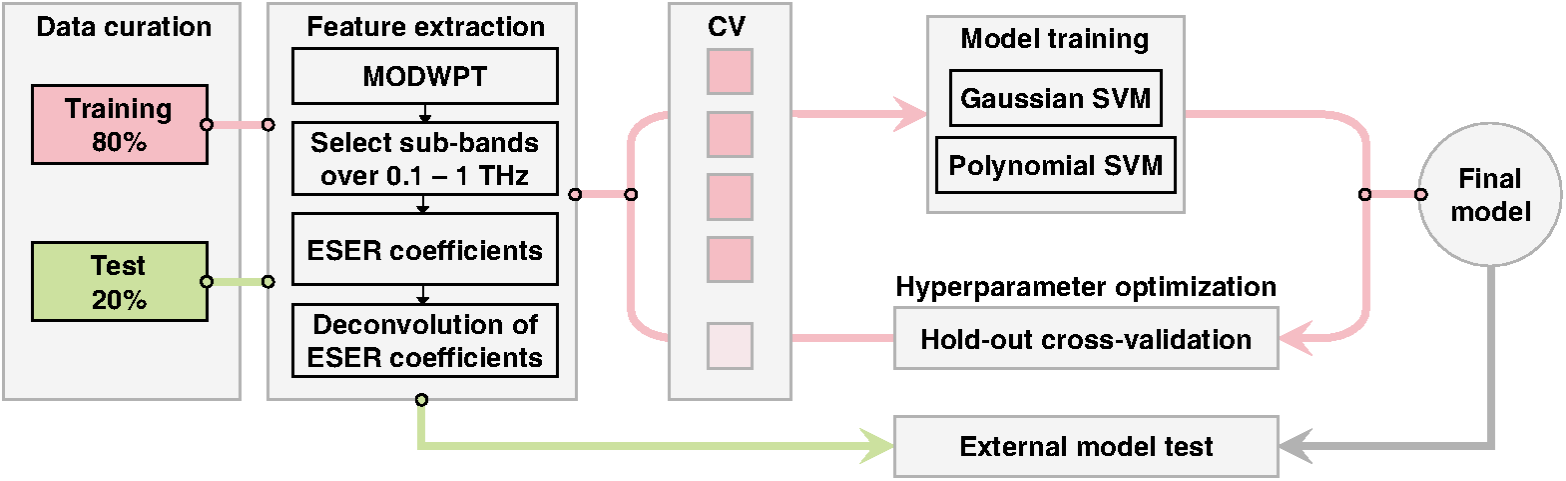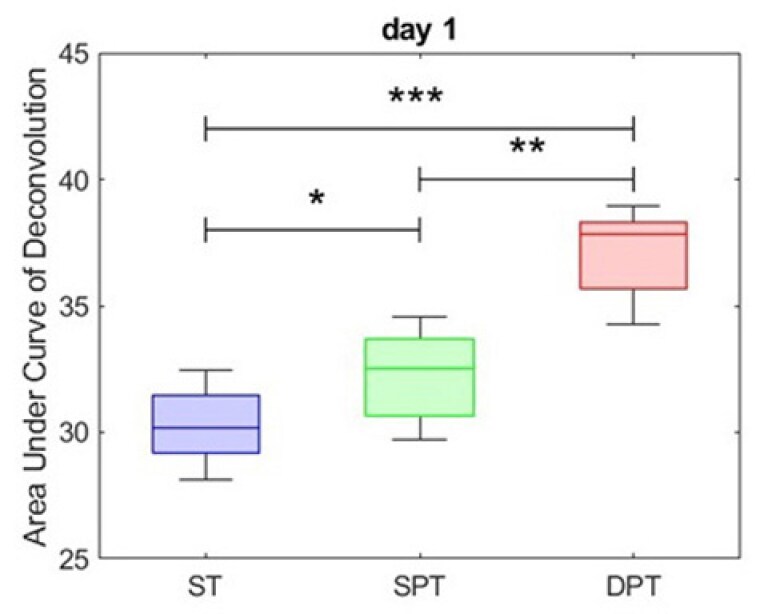# 514 Physics-based Deep Learning Models for Accurate Triage of Burn Wounds Using a Terahertz Spectral Scanner

**DOI:** 10.1093/jbcr/iraf019.143

**Published:** 2025-04-01

**Authors:** M Hassan Arbab, Zachery Harris, Adam Singer, Steven Sandoval

**Affiliations:** Stony Brook University; Stony Brook University; Stony Brook University; Stony Brook University

## Abstract

**Introduction:**

The formation of edema and the dynamic nature of the zone of stasis, surrounding the zone of coagulation, of a burn are mainly responsible for inaccuracies in burn delineation. Today, burn triage is still based on visual and tactile inspection by experienced surgeons, while histology remains the gold standard, albeit invasive and time-consuming. The complexity of the dynamic molecular and cellular level changes, which skin constituents experience post burn, gives rise to most of the discrepancies in burn assessment. Early and highly accurate differentiation of burn wounds can alter the treatment course, reduce length of hospital stay and improve overall recovery of the patients. Terahertz spectroscopy is a promising new technology that can differentiate between burn wounds by quantifying the bound and free water content of the tissue as well as the scattering by deep dermal structures.

**Methods:**

Recently, physics-based deep learning models to predict the healing outcomes of porcine burns have exploited the rich terahertz spectral data to achieve highly accurate classification on Day 1 after injury. Using a Support Vector Machine and Deep Neural Networks an accuracy between 90 to 94.7% was achieved to predict if the burn would re-epithelialize spontaneously within 28 days. In this presentation, we explore the utility of the same AI models and the terahertz handheld scanner in the first pilot human study of this technology. We monitored the healing outcome of patients (n = 20 burns) admitted within 48 hours of the initial injury. If the attending physician determined that surgical intervention was necessary, we obtained histological biopsies from the excised tissue to determine the depth of the burn (control experiment). However, if the burn was determined to be superficial partial thickness, we monitored the re-epithelialization rate weekly (on days 7, 14, 21, and 28) to determine the wound closure date, which serves as the ground truth for the machine learning algorithm. As shown in the attached figure, in five-fold cross-validation, a model is first trained over the training set (80% of spectral data), and the remaining 20% is reserved for calculating the classification error. We calculate the sensitivity, specificity, and accuracy rates, using receiver operating characteristic (ROC) analysis.

**Results:**

Preliminary results from this ongoing pilot clinical study indicate that the terahertz spectroscopy can achieve similarly high accuracy results (>90%) in predicting the healing outcome of burn wounds.

**Conclusions:**

This presentation will report on the first use of the terahertz spectral imaging modality in a pilot human study. Our preliminary results indicate that terahertz spectroscopy can achieve high accuracy in differentiating burn wounds on Day 1 post-injury and predict the ultimate healing outcome.

**Applicability of Research to Practice:**

An accurate and precise method of burn depth classification is essential for making appropriate burn treatment decisions.

**Funding for the Study:**

U.S. Army Medical Research Acquisition Activity (USAMRAA) through the Military Burn Research Program (MBRP) and the National Institute of General Medical Sciences (NIGMS) of the National Institutes of Health.